# Immunohistochemical expression of Cyclin D1 among Sudanese patients diagnosed with benign and malignant prostatic lesions

**DOI:** 10.1186/s13104-020-05138-7

**Published:** 2020-06-17

**Authors:** Eiman Siddig Ahmed, Lubna S. Elnour, Rowa Hassan, Emmanuel E. Siddig, Mintu Elsa Chacko, Eman T. Ali, Mona A. Mohamed, Abdalla Munir, Mohamed S. Muneer, Nouh S. Mohamed, Ali M. M. Edris

**Affiliations:** 1 Mycetoma Research Center, University of Khartoum, Khartoum, Sudan; 2 Department of Cytology and Histopathology, Faculty of Medical Laboratory Sciences, University of Khartoum, Khartoum, Sudan; 3 Nile University– School of Medicine, Khartoum, Sudan; 4 Department of Histopathology and Cytology, Alfarrabi College for Science and Technology, Khartoum, Sudan; 5 Faculty of Medicine, National University, Khartoum, Sudan; 6 Department of Histopathology and Cytology, Faculty of Medical Laboratory Sciences, National University, Khartoum, Sudan; 7 Department of Parasitology and Medical Entomology, Faculty of Medical Laboratory Sciences, Nile University, Khartoum, Sudan; 8 Department of Bioinformatics and Biostatistics, National University Research Institute, National University, Khartoum, Sudan; 9 Department of Neurology, Mayo Clinic, Jacksonville, FL USA; 10 Department of Radiology, Mayo Clinic, Jacksonville, FL USA; 11 Department of Internal Medicine, Faculty of Medicine, University of Khartoum, Khartoum, Sudan; 12 Department of Parasitology and Medical Entomology, Faculty of Medicine, Sinnar University, Sinnar, Sudan; 13 Molecular Biology Department, Alfarrabi College for sciences and Technology, Khartoum, Sudan; 14 Department of Histopathology and Cytology, Faculty of Applied Medical Sciences, University of Bisha, Bisha, Kingdom of Saudi Arabia

**Keywords:** Prostate cancer, Immunohistochemical expression, Prognostic markers, Cyclin D1

## Abstract

**Objectives:**

Prostate cancer (PC) is common cancer worldwide. Several markers have been developed to differentiate between benign prostatic hyperplasia (BPH) from PC. A descriptive retrospective hospital-based study aimed at determining the expression of Cyclin D1 in BPH and PC. The study took place at different histopathology laboratories in Khartoum state, Sudan, from December 2016 to January 2019. Formalin-fixed paraffin-embedded blocks were sectioned and fixed in 3-aminopropyltriethoxysilane coated slides incubated into primary antibody for Cyclin D1. The assessment of immunoreactivity of Cyclin D1 of each section was done using the Gleason scoring system.

**Results:**

A total of 153 males’ prostate sections included in this study, of them, 120 (78.4%) were PC, and 33 (21.6%) were BPH. Their age ranged from 45 to 88 years, mean age was 66.19 ± 8.599. 142 (92.8%) did not have a family history of PC, while 11 (7.2%) patients reported having a family history. The Gleason scoring showed a total of 81 (52.9%) patients with high-grade and 39 (25.5%) with low-grade. 118 (97.5%) patients had PC showed positive results for Cyclin D1, while BPH was 3 (2.5%). P value < 0.001. Cyclin D1 staining was associated with high-grade Gleason score and perineural invasion, P value 0.001.

## Introduction

Prostate cancer (PC) is considered as the third most common type of cancer worldwide and the second most cancer among males with estimated 1,276,106 PC cases in 2018 with 358,989 deaths worldwide [1]. Consequently, several markers have been developed over the last years to differentiate between benign prostatic hyperplasia (BPH) from PC [2, 3].

Cyclin D1 is a member of proteins that belong to D-type Cyclin family; those are involved in the regulation of cell cycles by mediating the phosphorylation and inactivation of the retinoblastoma protein, allowing the cells to progress from G1 phase to S phase [4]. High expression of Cyclin D1 was noticed among different types of malignancy, including Breast cancer [4], colon cancer [5], and lung cancer [6, 7]. There are many studies that investigated the role of Cyclin D1 expression in PC; Pereira et al., correlated the expression of Cyclin D1 to be associated with perineural invasions and with the aggressive form of the disease [8].

In Sudan, PC is one of the cancers that always being lately presented [9], since most of the patients are suffering from stigmatization and poverty, this might cause the inability of the patient to early screen for the disease as well as continue to follow up to ensure adequate treatment and avoid depraved prognosis to increase life expectancy [10]. During 2009–2010, a total of 6771 new cancer cases were registered, of them, 387 (%) were PC cases [10]. Also in 2009-2013, PC was one of the top 5 prevalent cancers in Sudan, about 2000 PC cases were reported in that period with a 10% mortality rate [10, 11]. In Sudan, scarce data about the expression level of Cyclin D1 among Sudanese PC patients, as this is attributed to the lack of expression studies [9]. Therefore, not only investigation towards appropriate marker can be used to predict the prognosis of PC among males in Sudan, but also will lead for a better patients’ management and upcoming with efficient follow up strategies. Hence, in this study, we aimed to investigate the Cyclin D1 expression in patients with BPH and PC and to correlate the expression with the clinical characteristics of the patients, and to emphasize the use of Cyclin D1 as a diagnostic and prognostic marker for PC in Sudan.

## Main text

### Materials and methods

A descriptive retrospective hospital-based study conducted at two histopathology laboratories; the Military Hospital and Soba teaching hospital, in Khartoum state-Sudan, from December 2016 to January 2019. The study samples included 153 formalin fixed paraffin embedded blocks from patients diagnosed with BPH (n = 33) and PC (n = 120).

#### Preparation of slides toward immunohistochemistry

From each paraffin embedded block, two sections were cut. One section was stained using hematoxylin and eosin technique (H&E). The second section was mounted onto 3-aminopropyltriethoxysilane coated slides (LabScientific, NJ-USA). Antigen retrieval was performed by treating the section in citrate buffer incubated in a water bath at 96º C for 10 min. Then, slides were rinsed in distilled water and treated with 3% hydrogen peroxide in a methyl alcohol solution for 15 min. After that, slides were rinsed in a washing buffer for 5 min. The sections were then incubated in the primary antibody (rabbit monoclonal antibody to Cyclin D1, clone EPR2241, Biogenex, CA-USA) at room temperature for 2 h. After that, the staining was performed according to manufacturer protocol. For the positive control, a section from a known colon cancer tissue block was used. Whereas, for the negative control, another tissue section was used without applying the primary antibody.

#### Assessment of immunoreactivity of Cyclin D1

The assessment of immunoreactivity of Cyclin D1 was done using the Gleason scoring system [12]; 1 + if 10% of the cells expressed the marker, 2 + if > 10 – 25% of the cells are expressing the marker, 3 + if > 25 – 75% of the cells showed expression of the marker and 4 + if more than 75% of the cells expressed the marker. 1 + and 2 + were considered as low-grade, while 3 + and 4 + were considered as high-grade expression. Gleason scoring for grade 1 and 2 when simple round glands, close-packed or loosely packed in vague in rounded masses with well- or loosely defined edges, and grade 3; glands appear with varying small sizes irregular shape and irregular spacing with infiltrating edges. Whereas, grade 4; when glands sizes were small, medium, or large and fused into cords or ragged, with infiltrating masses.

### Statistical analysis

Data were analyzed using the Statistical Package for Social Sciences (SPSS) version 16. Pearson correlation with 95% confidence interval was used to test the association of Cyclin D1 expression with age. Chi Square test was done to test the significance of age with diagnosis and Cyclin D1 grade with the diagnosis categories. ANOVA test were done to test the association of age with family history, perineural invasion and angiolymphatic invasion. P value < 0.05 was considered a statistically significant.

### Results

#### Study characteristics

A total of 153 male participated in this study, of them, 120 (78.4%) were PC patients, and 33 (21.6%) were BPH. Age of the study participants ranged from 45 to 88 years, with a mean age of 66.19 ± 8.599. Among the 153 patients, 142 (92.8%) did not have a family history of PC, while 11 (7.2%) patients were reported to have a family history of PC.

#### Analysis results based on participant’s age

No association for age with Gleason score, family history, perineural invasion, angiolymphatic invasion and diagnosis (P values; 0.957, 0.110, 0.187, 0.466, 0.933 and 0.853 respectively). However, Gleason scoring were found to be positively correlated with patients age (P value = 0.711, Person’s r = 0.030).

#### Analysis based on age grouping

Participants were grouped into 4 groups according to their age group; a group of less than 60 years consisted of 29 (19.0%) patients, 60–69 years age group consisted of 74 (48.4%) patients, 70–79 years age group composed of 38 (24.8%) and those who are aged 80 years and more than 80 were 12 (7.8%) patients. Of the 120 patients diagnosed with PC, the age range of 60–69 years was the most frequent age group with a frequency of 48.3%.

No statistically significant difference was found in the comparison of diagnosis based on the age group, P value 0.798. A total of 62 (40.5%) were found to have a perineural invasion among the age group of 60-69 years (45.2%).

Association between age groups and frequency of perineural invasion was found to be statistically not significant, P value 0.396. For the angiolymphatic invasion, a total of 12 (7.8%) were reported. No statistically significant difference for the age group with the frequency of angiolymphatic invasion was found (P value 0.621). However, Cyclin D1 was found to be positive among a total of 121 (79.1%) patients, with the age group 60–69 years.

High-grade Gleason scores were mostly reported among 60–69 years age group; 38 (46.9%) No statistically significant difference found for Gleason scoring based on age grouping (P value 0.915). Based on the family history of the patient with PC, 142 (92.8%) of study patients did not have any family history of PC, P value 0.361 (Additional file 1).

#### Analysis based on diagnosis

**Fig. 1:**
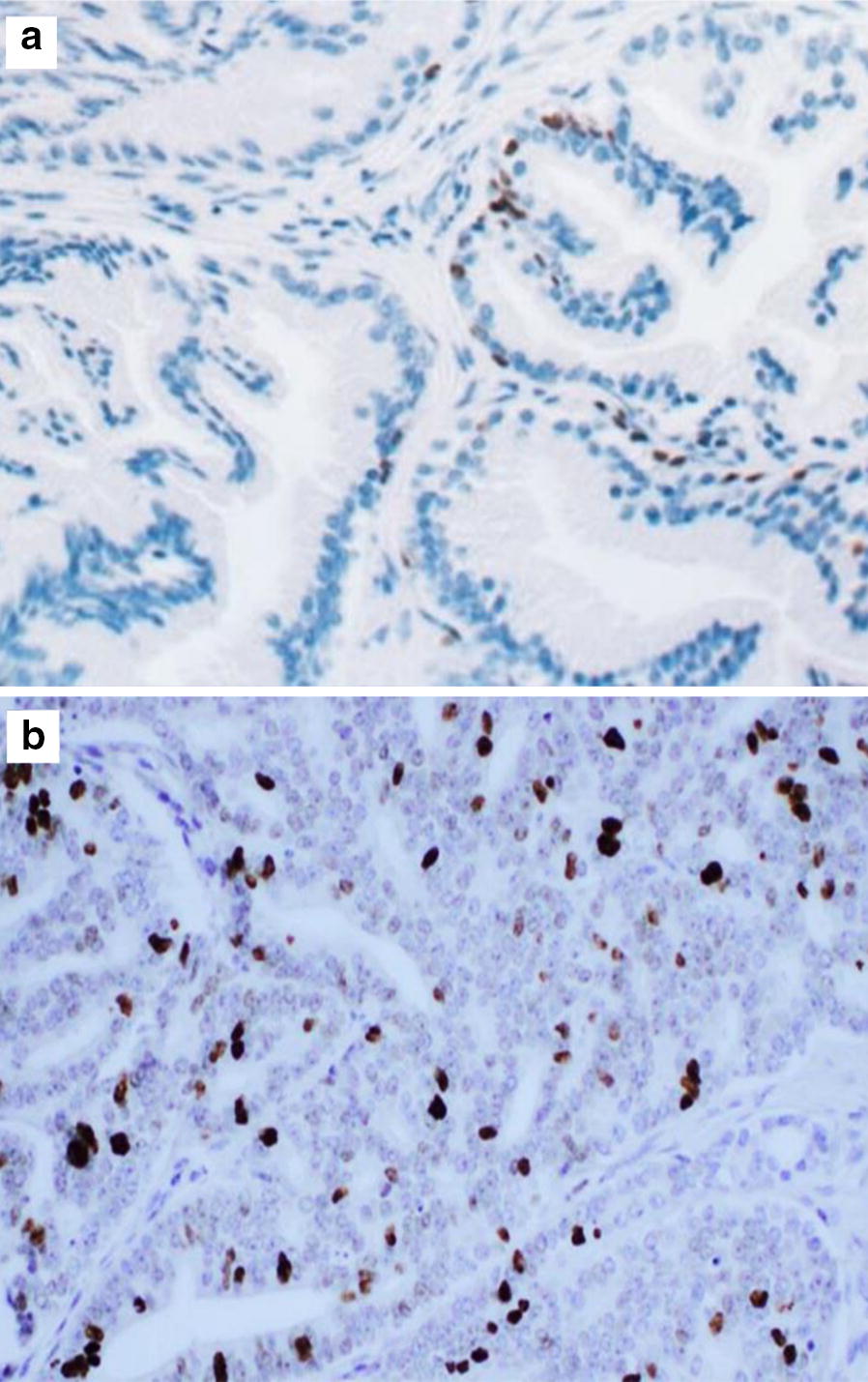
The correlation of Cyclin D1 immunohistochemistry expression in BPH and PC. **a** shows the level of expression of cyclin D1 in benign prostatic hyperplasia (BPH), **b** shows the high expression level of Cyclin D1 in prostate cancer (PC)

Of the total, 121 (79.1%) patients showed positive results for Cyclin D1 stain, the highest frequency was among patients diagnosed as PC; 118 (97.5%). While, BPH showed minimal frequency for Cyclin D1 positivity; 3 (2.5%). Positive Cyclin D1 was highly significant with PC (P value < 0.001) (Fig. 1).

Patient’s family history was not associated with having PC or BPH (P value 0.062). Gleason Scoring system showed presence of 39 (25.5%) patients with low-grade and 81 (52.9%) with high-grade expression.

#### Analysis based on Cyclin D1 staining results

**Fig. 2:**
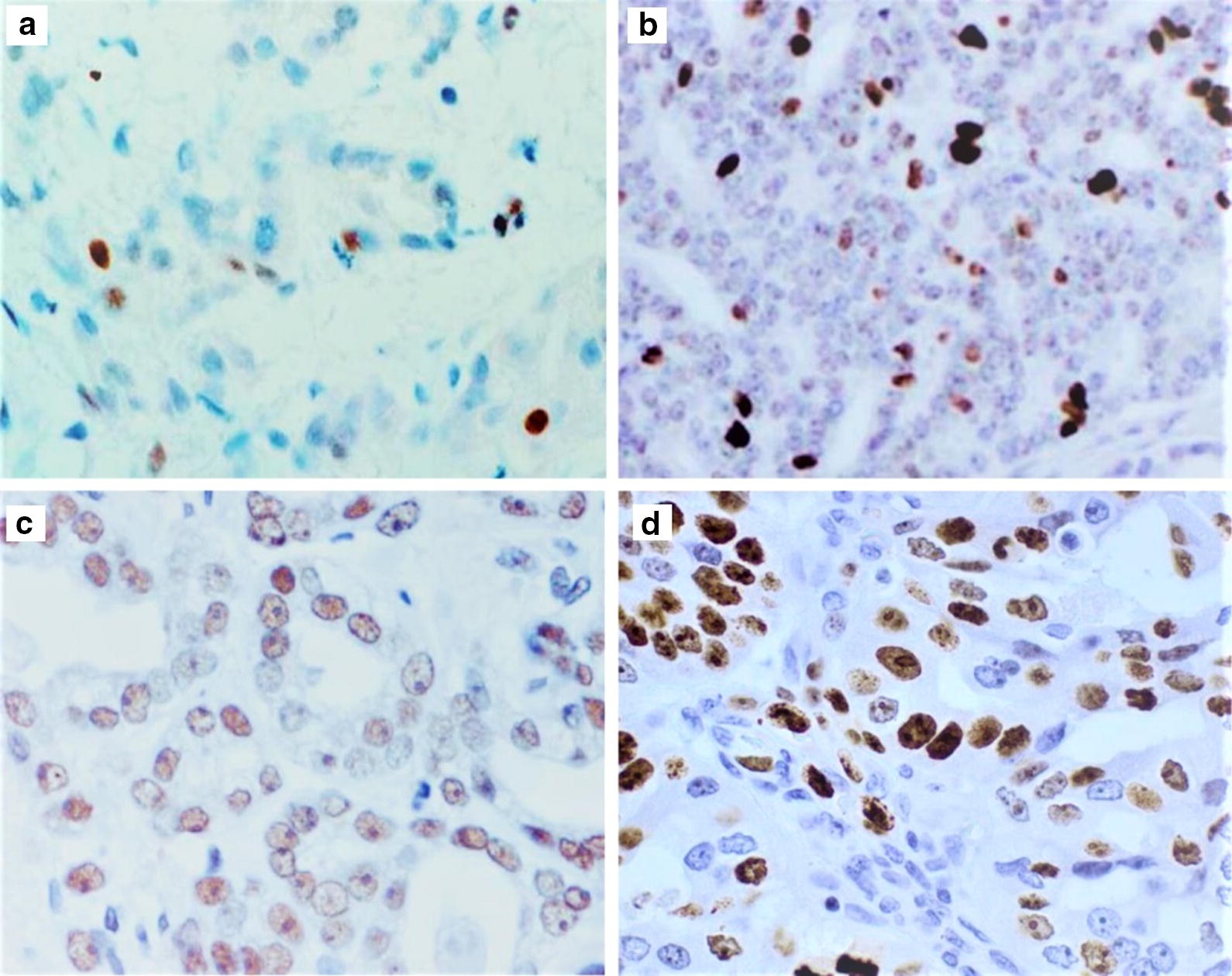
Photomicroscopy from representative Cyclin D1 immunohistochemistry expression; **a** and **b** indicates low-grade expression; 10% to 25% of the cells were expressing the Cyclin D1. **c** and **d** indicates high-grade expression, in C: (> 25–75%) of the cells showed expression of the marker. in **d** more than 75% of the cells expressed the marker

Of the 153 Patients, 81 (100%). While for those with low-grade Gleason score, 37 (94.9%) were positively stained. However, 3 (9.1%) of the BPH were stained positive with Cyclin D1 (Fig. 2). Staining with Cyclin D1 was highly associated with high Gleason Score (P value < 0.001).

Results of Cyclin D1 staining were statistically insignificant among patients with family history of PC, although all participants with a family history; 11(5.2%) were positively stained with Cyclin D1, P value 0.068.

All patients having perineural invasion; 62 (40.5%) of them were positively stained with Cyclin D1. Staining with Cyclin D1 was also highly associated with perineural invasion (P value 0.000). On the other hand, staining with Cyclin D1 did not have a statistically significant difference, for patients who had angiolymphatic invasion, P value 0.053 (Table 1).

**Table 1:** Analysis based on Cyclin D1 staining results

	Cyclin D1 staining result		Total	*P* value
	Negative staining	Positive staining		
Gleason score				
Low-grade	2 (5.1%)	37 (94.9%)	39 (25.5%)	0.000
High-grade	0 (0.0%)	81 (100%)	81 (52.9%)	
Normal	30 (90.9%)	3 (9.1%)	33 (21.6%)	
Family history of PC				
No	32 (22.5%)	110 (77.5%)	142 (92.8%)	0.068
Yes	0 (0.0%)	11 (100%)	11 (7.2%)	
Perineural invasion				
No	32 (35.2%)	59 (64.8%)	91 (59.5%)	0.000
Yes	0 (0.0%)	62 (100%)	62 (40.5%)	
Angiolymphatic invasion				
No	32 (22.7%)	109 (77.3%)	141 (92.2%)	0.053
Yes	0 (0.0%)	12 (100%)	12 (7.8%)	
Diagnosis^a^				
BPH	30 (90.9%)	3 (9.1%)	33 (21.6%)	0.000
PC	2 (1.7%)	118 (98.3%)	120 (78.4%)	

^a^*BPH* Benign prostatic hyperplasia, *PC* Prostate cancer

The distribution of Cyclin D1 staining results, angiolymphatic invasion, perineural invasion, family history, and Gleason score among BPH and PC patients is described in Additional file 2.

### Discussion

PC is considered as the most prominent malignancy encountered in male, especially those above 60 years of age. Prostate malignancy has a characteristic of late presentation and lousy prognosis, especially among Sudanese populations [9]. Although different molecular markers have been used to enable the differentiation between benign and malignant lesions and to predict the prognosis [13–19], varied results were obtained for these markers, in which some were involved in PC development, such as Cyclin D1 which increases its expression in cases of metastasis development [16], and it was correlated with poor prognosis in tumor cells of the breast, pancreas, esophageal carcinoma, and mantle cell lymphoma [20–23]. Cyclin D1 overexpression has reflected the aggressiveness, recurrence, and shortening of patient life expectancy [24, 25]. In this study, since most of the BPH tissues did not show high expression level, although there was a report indicated that the increase in expression of Cyclin D1 is usually rare among PC [26].

In respect to demographical data of patients, the vast majority of PC patients are those between the 5th and 7th decade of their life, and those with a family history of PC. In this study, the reported Cyclin D1 expression was not association with age or patient’s family history as reported previously [27]. Even though it is in contrast with the earlier study conducted by Dunsmuir et al., they found an association between age and Cyclin D1 expression [28].

Correspondingly to previous reports [8, 29–32], that stated a significant association especially with high-grade tumors, in this study the reported correlation of Cyclin D1 and Gleason score was statistically significant (P value = 0,001). While in another study expression of Cyclin D1 in BPH was not detected [31], and in another study, although Cyclin D1 is expressed in BPH, rarely Cyclin D1 is overexpressed in cases of PC [26].

In this study, the relation between Gleason Scoring and Cyclin D1 expression were in harmony to previous reports, showing a positive relationship between the expression of Cyclin D1 and Gleason score, especially with high-grade tumors [8, 22, 29–31]. While in comparison to other studies, no association was found [29, 33, 34].

In this study, a significant association between the Cyclin D1 expression and the perineural invasion (P value < 0.001); this result agrees with that of Pereira et al. [8], and He et al. [35]. Their results showed that the overexpression of this marker besides other markers leads to the increase of cells proliferation and transformation. Since Cyclin D1 increases the mobility and invasion of tumor cells, the overexpression of Cyclin D1 is related to the aggressiveness of PC [35, 36]. Therefore, Cyclin D1 was expressed mostly among patients with perineural invasion. In some studies, Cyclin D1 expression was correlated with poor prognosis in the tumor cells [20–23], and it is overexpression has reflected the aggressiveness of cancer [24, 25]. As well, due to the predictive preoperative usefulness of the Gleason score correlated with Cyclin D1 expression to predict tumor behavior, it became easy to differentiate between malignant and benign disease [37, 38]. However, in some studies, Gleason score did not satisfy that theme [8, 39–41].

### Conclusion

The use of Cyclin D1 expression immunohistochemical detection system is a very suitable way for diagnosing PC, especially in low-income settings such as Sudan. Also, Cyclin D1 can also be used as indicative for PC progression and predicting tumor cells invasion to perineural tissues.

## Limitations

- The sample size of this study, although it provided some clues on the expression of Cyclin D1 in Sudanese PC patients, a more extensive study scale with an appropriate follow up of the PC progression would allow these results to be sensible.
- Failure to obtain follow up data of the PC patients reviewed in this study prevent the investigation about the relationship of other PC progressions with Cyclin D1 expression.

## Supplementary information

**Additional file 1: Table S1.** Analysis based on age grouping.

**Additional file 2: Table S1.** The distribution of Cyclin D1 staining results, angiolymphatic invasion, perineural invasion, family history, and Gleason score among BPH and PC patients.

## Data Availability

The datasets used and/or analyzed during the current study are available from the corresponding author on reasonable request.

## Abbreviations

BPH: Benign prostatic hyperplasia
H&E: Hematoxylin and Eosin
PC: Prostate cancer
