# Supplementary material for: Immunohistochemical expression of Cyclin D1 among Sudanese patients diagnosed with benign and malignant prostatic lesions
Source: BMC Res Notes. 2020 Jun 17;13:295. doi: 10.1186/s13104-020-05138-7 (PMC7302005; doi:10.1186/s13104-020-05138-7)
Supplement: Supplementary file 1 — Additional file 1: Table S1. Analysis based on age grouping. [file 13104_2020_5138_MOESM1_ESM.docx]

**Additional file 1:**

**Table S1:** Analysis based on age grouping.

|  | **Patients Age groups** | | | | **Total** | **P value** |
| --- | --- | --- | --- | --- | --- | --- |
|  | **less than 60 years** | **60-69 years** | **70-79 years** | **80 years and more** |  |  |
| **Diagnosis*** | | | | | | |
| BPH | 8 (24.2%) | 16 (48.5%) | 7 (21.2%) | 2 (6.1%) | 33 (21.6%) | 0.798 |
| PC | 21 (17.5%) | 58 (48.3%) | 31 (25.8%) | 10 (8.3%) | 120 (78.4%) |  |
| **Perineural invasion** | | | | | | |
| No | 14 (15.4%) | 46 (50.5%) | 22 (24.2%) | 9 (9.9%) | 91 (59.5%) | 0.396 |
| Yes | 15 (24.2%) | 28 (45.2%) | 16 (25.8%) | 3 (4.8%) | 62 (40.5%) |  |
| **Angiolymphatic invasion** | | | | | | |
| No | 26 (18.4%) | 69 (48.9%) | 34 (24.1%) | 12 (8.5%) | 141 (92.2%) | 0.621 |
| Yes | 3 (25.0%) | 5 (41.7%) | 4 (33.3%) | 0 (0.0%) | 12 (7.8%) |  |
| **Cyclin D1** | | | | | | |
| Negative | 7 (21.9%) | 16 (50.0%) | 7 (21.9%) | 2 (6.3%) | 32 (20.9%) | 0.924 |
| Positive | 22 (18.2%) | 58 (47.9%) | 31 (25.6%) | 10 (8.3%) | 121 (79.1%) |  |
| **Gleason score** | | | | | | |
| Low-grade | 7 (17.9%) | 20 (51.3%) | 8 (20.5%) | 4 (10.3%) | 39 (25.5%) | 0.915 |
| High-grade | 14 (17.3%) | 38 (46.9%) | 23 (28.4%) | 6 (7.4%) | 81 (52.9%) |  |
| Normal | 8 (24.2%) | 16 (48.5%) | 7 (21.2%) | 2 (6.1%) | 33 (21.6%) |  |
| **Family history of PC** | | | | | | |
| No | 25 (17.6%) | 70 (49.3%) | 35 (24.6%) | 12 (8.5%) | 142 (92.8%) | 0.361 |
| Yes | 4 (36.4%) | 4 (36.4%) | 3 (27.3%) | 0 (0.0%) | 11 (7.2%) |  |

*BPH; Benign prostatic hyperplasia, PC; Prostate Cancer.
