# Supplementary material for: Immunohistochemical expression of Cyclin D1 among Sudanese patients diagnosed with benign and malignant prostatic lesions
Source: BMC Res Notes. 2020 Jun 17;13:295. doi: 10.1186/s13104-020-05138-7 (PMC7302005; doi:10.1186/s13104-020-05138-7)
Supplement: Supplementary file 2 — Additional file 2: Table S1. The distribution of Cyclin D1 staining results, angiolymphatic invasion, perineural invasion, family history, and Gleason score among BPH and PC patients. [file 13104_2020_5138_MOESM2_ESM.docx]

**Additional file 2:**

**Table S1:** The distribution of Cyclin D1 staining results, angiolymphatic invasion, perineural invasion, family history, and Gleason score among BPH and PC patients

|  | **Diagnosis*** | | **Total** | **P value** |
| --- | --- | --- | --- | --- |
|  | **BPH** | **PC** |  |  |
| **Cyclin D1** | | |  |  |
| Negative | 30 (93.8%) | 2 (6.3%) | 32 (20.9%) | 0.000 |
| Positive | 3 (2.5%) | 118 (97.5%) | 121 (79.1%) |  |
| **Angiolymphatic invasion** | | |  |  |
| No | 33 (23.4%) | 108 (76.6%) | 141 (92.2%) | 0.048 |
| Yes | 0 (0.0%) | 12 (100%) | 12 (7.8%) |  |
| **Perineural invasion** | | |  |  |
| No | 33 (36.3%) | 58 (63.7%) | 91 (59.5%) | 0.000 |
| Yes | 0 (0.0%) | 62 (100%) | 62 (40.5%) |  |
| **Family History of PC** | | |  |  |
| No | 33 (23.2%) | 109 (76.8%) | 142 (92.8%) | 0.062 |
| Yes | 0 (0.0%) | 11 (100%) | 11 (7.2%) |  |
| **Gleason score** | | |  |  |
| Low-grade | 0 (0.0%) | 39 (100%) | 39 (25.5%) | 0.000 |
| High-grade | 0 (0.0%) | 81 (100%) | 81 (52.9%) |  |
| Normal | 33 (100%) | 0 (0.0%) | 33 (21.6%) |  |

*BPH; Benign prostatic hyperplasia, PC; Prostate Cancer.
